# Assessing the impact of comorbidities on surgical outcomes in laparoscopic gastrectomy

**DOI:** 10.1016/j.sopen.2025.12.003

**Published:** 2025-12-18

**Authors:** Saleha Khan, Aleena Zobairi, Ibrahim Kutbi, Nuran Alsobyani, Hamad Aldobashi, Zulaihat Galadima, Ameena Manzoor, Sjaak Pouwels

**Affiliations:** a College of Medicine and Surgery, Batterjee Medical College, Jeddah, Saudi Arabia; b Department of Surgery, Bielefeld University – Campus Detmold, Klinikum Lippe, Detmold, NRW, Germany; c Department of Intensive Care Medicine, Elisabeth-Tweesteden Hospital, Tilburg, the Netherlands

**Keywords:** Laparoscopic gastrectomy, Comorbidity, Complications, Gastrectomy, Gastric Cancer

Dear editor,

Laparoscopic gastrectomy (LG) has emerged as a cornerstone in the surgical management of gastric cancer, particularly in its early stages. Compared to conventional open surgery, LG offers numerous advantages, including reduced postoperative pain, shorter hospital stays, faster recovery, and improved cosmetic outcomes, contributing to its widespread use [1]. Despite these advancements, the presence of comorbidities continues to pose significant challenges to achieving optimal outcomes in patients undergoing LG. Comorbid conditions such as cardiovascular disease, diabetes mellitus, respiratory disorders, liver disease, and obesity have been increasingly recognized as key determinants of surgical outcomes [2]. The interplay between comorbidities and other patient-specific factors, such as age and body mass index (BMI), adds further complexity to perioperative management. This exacerbates perioperative risks, increases intraoperative complications, and prolongs recovery times. These findings underscore the necessity of comprehensive preoperative evaluation and tailored perioperative management to optimize surgical outcomes [2]. Minimally invasive approaches like LG have been shown to mitigate some of the risks associated with traditional open gastrectomy, particularly for patients with obesity. For instance, LG results in lower postoperative morbidity and shorter hospital stays compared to open procedures, even in patients with multiple comorbidities or severe obesity [3]. Given the increasing global life expectancy, the prevalence of patients with comorbidities requiring gastric cancer surgery is expected to rise. Countries such as Japan and Korea, where early gastric cancer is commonly detected through mass screening programs, are likely to see more elderly patients undergoing LG. Older patients undergoing LG face additional risks due to diminished physiological reserves and the cumulative burden of comorbid conditions. This heightened vulnerability not only increases the likelihood of complications but also prolongs recovery times, necessitating individualized surgical planning and meticulous postoperative care [4]. For these patients, factors such as preoperative optimization, surgical technique selection, and postoperative care protocols tailored to their comorbidity profiles are critical to improving outcomes. While the advantages of LG in reducing surgical trauma and facilitating recovery are well-documented, there remains a pressing need to comprehensively evaluate the influence of comorbid conditions on its outcomes. Existing literature suggests that comorbidities impact both intraoperative metrics, such as blood loss and operative time, and postoperative outcomes, including complications, hospital stay length, and long-term survival [5]. However, variability in study design, patient populations, and reported outcomes has limited the generalizability of findings. When searching in the literature (see our protocol registered in the International Prospective Register of Systematic Reviews (PROSPERO) under the ID [CRD42025640502]), there is very high heterogeneity in the current literature about the impact of comorbidities in gastric cancer surgery (irrespective of technique used.) Especially elderly patients (aged >65) represent a particularly vulnerable group. Age alone contributes to elevated surgical risk, but when compounded by coexisting comorbidities, the incidence of postoperative complications and prolonged recovery is notably higher [6]. As a result, comprehensive geriatric assessments and individualized perioperative strategies are essential for optimizing outcomes in this population.

## CRediT authorship contribution statement

**Saleha Khan:** Writing – review & editing, Writing – original draft, Visualization, Formal analysis, Data curation, Conceptualization. **Aleena Zobairi:** Writing – review & editing, Writing – original draft, Visualization, Formal analysis, Data curation, Conceptualization. **Ibrahim Kutbi:** Writing – review & editing, Writing – original draft, Visualization, Formal analysis, Data curation, Conceptualization. **Nuran Alsobyani:** Writing – review & editing, Writing – original draft, Visualization, Formal analysis, Data curation, Conceptualization. **Hamad Aldobashi:** Writing – review & editing, Writing – original draft, Visualization, Formal analysis, Data curation, Conceptualization. **Zulaihat Galadima:** Writing – review & editing, Writing – original draft, Visualization, Formal analysis, Data curation, Conceptualization. **Ameena Manzoor:** Writing – review & editing, Writing – original draft, Visualization, Formal analysis, Data curation, Conceptualization. **Sjaak Pouwels:** Writing – review & editing, Writing – original draft, Visualization, Supervision, Formal analysis, Data curation, Conceptualization.

## Ethical approval statement

This article did not involve any studies with human or animal subjects, and therefore ethical approval was not required.

## Funding sources

This study was not funded.

## Declaration of competing interest

The authors have no conflict of interest to declare.

## References

[1] Sun J., Li J., Wang J., Pan T., Zhou J., Fu X. (2012). Meta-analysis of randomized controlled trials on laparoscopic gastrectomy vs. open gastrectomy for distal gastric cancer. Hepato-gastroenterology.

[2] Kim W., Song K.Y., Lee H.J., Han S.U., Hyung W.J., Cho G.S. (2008). The impact of comorbidity on surgical outcomes in laparoscopy-assisted distal gastrectomy: a retrospective analysis of multicenter results. Ann Surg.

[3] Quan Y., Huang A., Ye M., Xu M., Zhuang B., Zhang P. (2016). Comparison of laparoscopic versus open gastrectomy for advanced gastric cancer: an updated meta-analysis. Gastric Cancer.

[4] Wang J.F., Zhang S.Z., Zhang N.Y., Wu Z.Y., Feng J.Y., Ying L.P. (2016). Laparoscopic gastrectomy versus open gastrectomy for elderly patients with gastric cancer: a systematic review and meta-analysis. World J Surg Oncol.

[5] Lei X., Wang Y., Shan F., Li S., Jia Y., Miao R. (2022). Short-and long-term outcomes of laparoscopic versus open gastrectomy in patients with gastric cancer: a systematic review and meta-analysis of randomized controlled trials. World J Surg Oncol.

[6] Song P., Tian W., Zhao J., Liu Y., Wang C., CAO L. (2026). Impact of Adjuvant Chemotherapy on Survival of the Patients with pT1N+Mo Gastric Cancer: A Retrospective Cohort Study. J Invest Surg.

